# Supplementary material for: Diagnostic Value of Chromosomal Microarray Analysis for Fetal Congenital Heart Defects with Different Cardiac Phenotypes and Extracardiac Abnormalities
Source: Diagnostics (Basel). 2023 Apr 20;13(8):1493. doi: 10.3390/diagnostics13081493 (PMC10137444; doi:10.3390/diagnostics13081493)
Supplement: Supplementary file 1 [file diagnostics-13-01493-s001.zip › diagnostics-2324255-supplementary.pdf]

Supplementary Material Table S1. Shows the details of pathogenic copy number variations in 38 CHD fetuses.

| case | Cardiac and Extra Cardiac Phenotype | Chr | pCNVs | Type | Size (Mb) | Syndromes | Major genes included | Pattern of Inheritance | Outcomes |
|---|---|---|---|---|---|---|---|---|---|
| 1 | VSD，endocardial fibroelastosis, MCDK | 1 | 849,466-5,747,184 | del | 4.8 | 1p36 deletion syndrome | *PRDM16, SKI* | / | TP, Autopsy [a] |
| 2 | TOF，FGR，limb abnormality | 5 | 36,069,681-40,557,872 | del | 4.4 | Cornelia de Lange | ***NIPBL*** | / | TP |
| 3 | VSD，spina bifida | 6 | 163,732,938-170,914,297 | del | 7.1 | 6q terminal deletion syndrome | ***DLL1*** | / | LTF |
| 4 | CoA，FGR | 7 | 72,653,992-74,154,209 | del | 1.5 | WBS | *ELN, LIMK1* | / | LTF |
| 5 | CAT, VSD, horseshoe kidney | 8 | 160,101-7,120,108 | del | 6.9 | | *CSMD1, ANGPT2* | / | TP, Autopsy [a] |
| 6 | HRHS，VSD，callosal dysplasia，SUA | 10 | 98,561,770-105,586,041 | dup | 7.0 | | *LBX1, BTRC, POLL* | / | TP, Autopsy [b1] |
| 7 | HLHS，hemivertebra | 11 | 12,674,297-13,486,847 | del | 8.8 | | ***FLI1*** | / | TP, Autopsy [a] |
| 8 | Single ventricle, DORV, PS | 11 | 81,354,834-106,214,708 | del | 24.8 | | *FZD4* | / | TP |
| 9 | Aortic stenosis, VSD, hydrocephalus | 12 | 103,403,037-133,777,562 | dup | 30.3 | Noonan syndrome 1 | ***PTPN11, TBX5*** | / | TP |
| 10 | VSD，CCAM | 16 | 15,438,164-18,172,468 | dup | 2.7 | 16p13.11 recurrent microduplication | *MYH11* | / | LTF |
| 11 | TOF，hyperechogenic bowel, ventriculomegaly | 17 | 16,736,262-20,417,235 | del | 3.6 | SMS | *RAI1* | / | TP, Autopsy [a] |
| 12 | VSD，osteogenic dysplasia | 17 | 14,077,970-15,482,833 | del | 1.4 | HNPP | *PMP22* | / | LTF |
| 13 | PA，VSD，RAA，choroid plexus cyst | 21 | 33,623,086-35,352,874 | del | 1.7 | Tokita-Kim syndrome | *RUNX1, SON, SYNJ1* | / | TP |

| | | | | | | | | | |
|---|---|---|---|---|---|---|---|---|---|
| 14 | VSD, PS, tricuspid valve dysplasia | 17 | 87,009–1,184,534 | del | 1.1 | MDLS | *LIS1* | / | TP，Autopsy [c] |
| | | 22 | 18,919,942–21,440,514 | del | 2.5 | 22q11.2 DS | *TBX1* | | |
| 15 | d-TGA, VSD | 22 | 18,901,424-21,790,211 | del | 2.8 | 22q11.2 DS | | / | TP |
| 16 | VSD, cor triatriatum, mitral valve dysplasia, SUA | 22 | 18,984,187-21,927,646 | dup | 2.9 | 22q11.2 microduplication syndrome | | / | TP，Autopsy [a] |
| 17 | TOF(APVS), VSD | 22 | 18,916,842-21,798,907 | del | 2.8 | 22q11.2 DS | | denovo | TP |
| 18 | VSD, aortic stenosis, hydramnios | 22 | 18,636,749-21,800,471 | del | 3.1 | 22q11.2 DS | | denovo | TP |
| 19 | TOF | 22 | 18,644,790-21,465,659 | del | 2.8 | 22q11.2 DS | | denovo | TP，Autopsy [c] |
| 20 | RAA，APLPA | 22 | 16,888,899-20,725,309 | del | 3.8 | 22q11.2 DS | | / | TP，Autopsy [a] |
| 21 | VSD, PS | 22 | 18,636,749-21,800,471 | del | 3.1 | 22q11.2 DS | | denovo | TP |
| 22 | VSD, CoA | 22 | 18,648,855-21,800,471 | del | 3.1 | 22q11.2 DS | | / | TP |
| 23 | DORV, VSD, PA | 22 | 18,939,748-21,721,712 | del | 2.7 | 22q11.2 DS | | denovo | TP，Autopsy [c] |
| 24 | RAA，VSD, left superior vena cava | 22 | 19,004,071-21,800,471 | del | 2.7 | 22q11.2 DS | | / | TP，Autopsy [a] |
| 25 | RAA，VSD | 22 | 18,901,201-21,480,190 | del | 2.5 | 22q11.2 DS | | / | TP |
| 26 | TOF | 22 | 18,906,341-21,460,123 | del | 2.5 | 22q11.2 DS | | / | TP，Autopsy [a] |
| 27 | TOF | 22 | 18,978,201-21,926,534 | del | 2.9 | 22q11.2 DS | | / | TP，Autopsy [a] |
| 28 | TOF, RAA，absence of ductus arteriosus | 22 | 18,636,750-21,800,471 | del | 3.1 | 22q11.2 DS | | / | TP，Autopsy [a] |
| 29 | VSD, CoA | 22 | 18,648,856-21,800,471 | del | 3.1 | 22q11.2 DS | | / | TP，Autopsy [b2] |
| 30 | TOF, RAA, thymus aplasia | 22 | 18,648,856-21,058,888 | del | 2.4 | 22q11.2 DS | | / | TP，Autopsy [a] |
| 31 | IAA, type A, aortic stenosis, ARSA | 22 | 18,648,856-21,800,471 | del | 3.1 | 22q11.2 DS | | / | TP，Autopsy [c] |
| 32 | TOF | 22 | 18,882,825-21,796,237 | del | 3.1 | 22q11.2 DS | | / | TP |
| 33 | TOF (PA-VSD) | 22 | 18,927,431-21,427,431 | del | 2.5 | 22q11.2 DS | | / | TP |
| 34 | Single ventricle, single atrium, CAT | 22 | 46933489-51219152 | del | 4.2 | PHMDS | *SHANK3* | denovo | TP |

| | | | | | | | | | |
|---|---|---|---|---|---|---|---|---|---|
| 35 | TAPVC, accessory ear, micrognathia | 22 | 16050230-18679614 | dup | 2.6 | CES | | / | TP，Autopsy [a] |
| 36 | RAA | X | 2,703,661-6,609,379 | del | 3.9 | | *ARSL* | denovo | Birth |
| 37 | TOF (PA-VSD), RAA, left superior vena cava, SUA | X | 168,552-55,508,714 | Del | 55.3 | LWD, Xq28 duplication | *SHOX, DMD,* | / | TP，Autopsy [a] |
| | | | 55,509,386-155,233,098 | dup | 99.7 | | *USP9X, GATA1, PIGA MECP2* | | |
| 38 | VSD, holoprosencephaly, facial abnormality | X | 134,743,723-155,043,723 | dup | 20.3 | Xq28 duplication | *MECP2* | / | TP |

MCDK，multicystic dysplastic kidney; SUA，single umbilical artery; CoA, coarctation of aorta; CAT, common arterial trunk; CCAM，congenital cystic adenomatoid malformation; APVS, absent pulmonary valve syndrome; APLPA, anomalous origin of left pulmonary from the ascending aorta; ARSA, aberrant right subclavian artery; DS: deletion syndrome; del: deletion; dup: duplication; WBS: Williams-Beuren Syndrome; SMS: Smith-Magenis syndrome; HNPP: Hereditary Liability to Pressure Palsies; MDLS: Miller-Dieker lissencephaly syndrome; PHMDS: Phelan-McDermid syndrome; CES: Cat eye syndrome; LWD: Leri-Weill dyschondrostsosis; LTF, lost to follow-up; TP, terminal pregnancy.

[a] autopsy results are consistent with prenatal ultrasound.

[b1] autopsy revealed repeated bladder and anal atresia, others were consistent with prenatal ultrasound.

[b2] autopsy result is IAA, type B.

[c] autopsy also found thymus hypoplasia/aplasia.

Bold font is the genes discussed in the article.

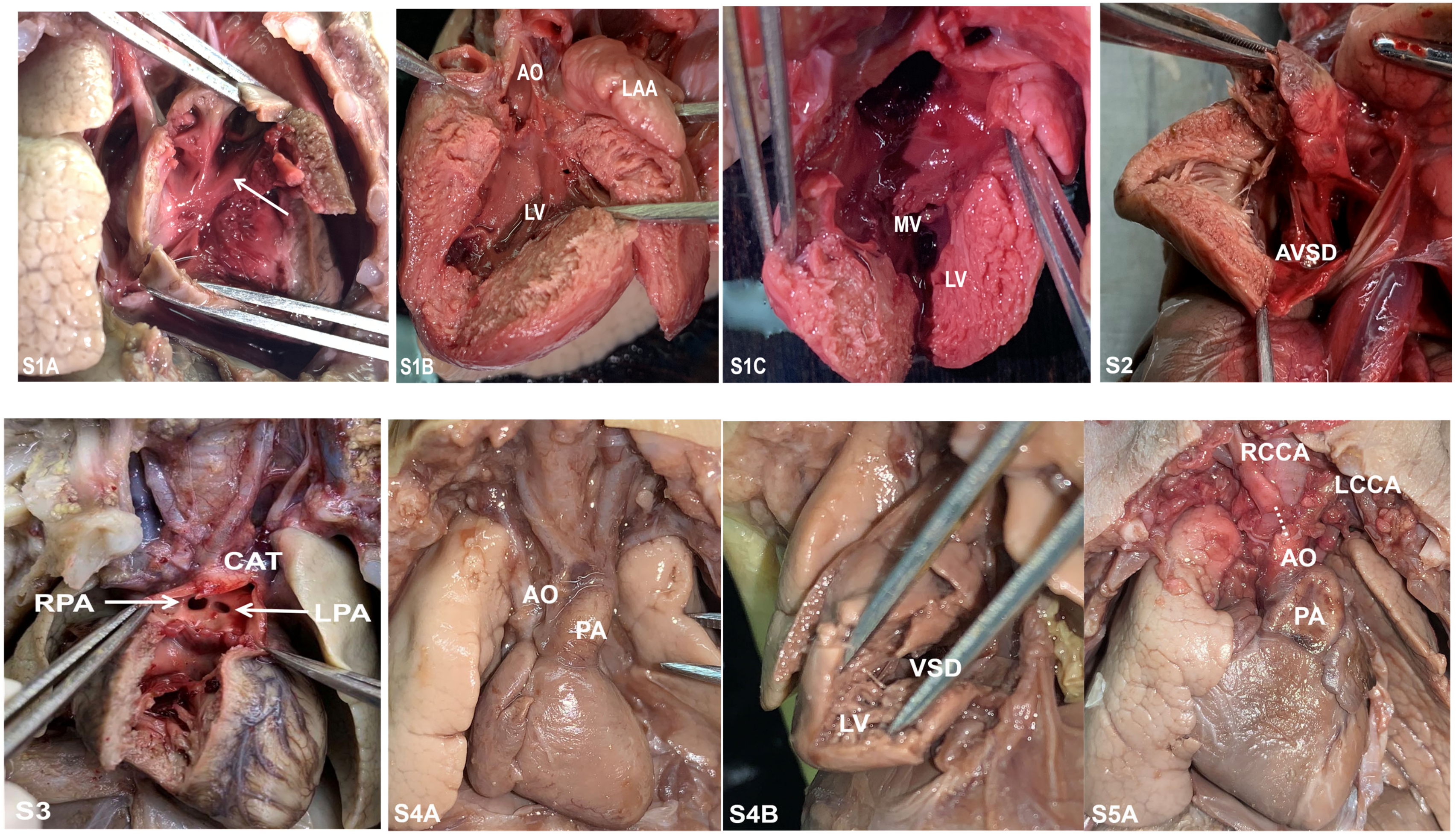

AO
LAA
LV
S1A
S1B
MV
LV
S1C
AVSD
S2
CAT
RPA
LPA
S3
AO
PA
S4A
VSD
LV
S4B
RCCA
LCCA
AO
PA
S5A

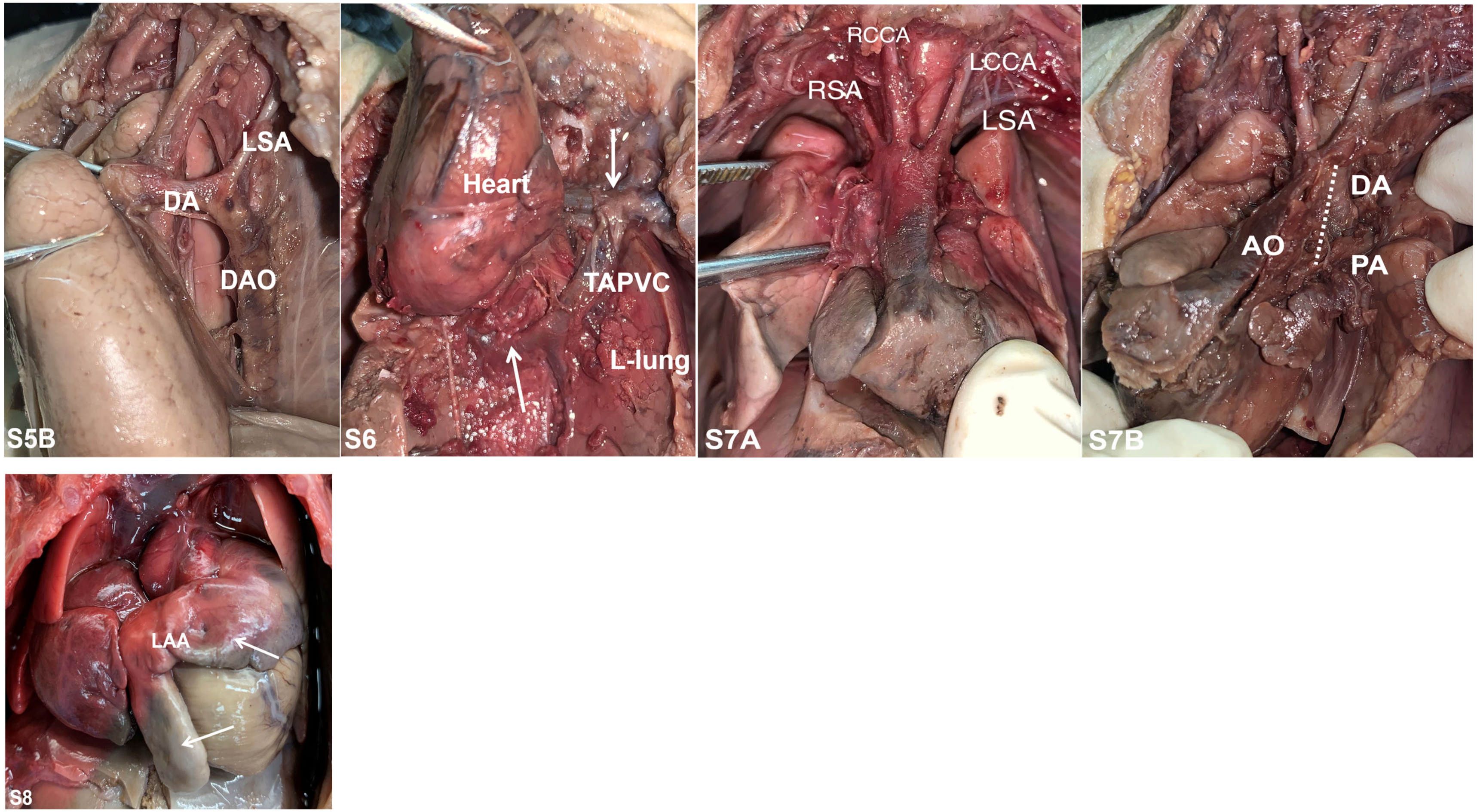


**Figure S1-S8 shows the autopsy of CHD fetus.**

Fig S1, mitral valve dysplasia; S1A, arrow shows no foramen ovale; S1B, LV and LAA thickening, stenosis aorta; S1C, mitral valve dysplasia, CNV result was VUS; Fig S2, complete type AVSD, trisomy 18; Fig S3 shows case 5 in table 2, autopsy results show CAT (type II); Fig S4 shows

case 7 in table 2; S4A, aorta dysplasia; S4B, LV is very small, VSD can be seen. Fig S5 shows case 29; S5A, only two blood vessels are sent out in ascending aorta; S5B, LSA is sent from descending Ao. Figure S6 shows case 35, and the arrows show common vena cava and vertical vein, respectively; Figure S7 shows case 37; S7A, RAA with mirror image branching; S7B, PA atresia, left and right pulmonary arteries supplied by DA. Dotted line, blood vessel accidentally broken; LAA, left atrial appendage; Ao, aorta; PA, pulmonary artery; LV, left ventricle; MV, mitral valve; CAT, common arterial trunk; LCCA, left common carotid artery; RCCA, right common carotid artery; LSA, subclavian artery; DA, ductus arteriosus. Fig S8, prenatal diagnosis of abnormal cardiac function, autopsy found a huge LAA, CNV result was VUS.
